# Ultrasmall and phase-pure W_2_C nanoparticles for efficient electrocatalytic and photoelectrochemical hydrogen evolution

**DOI:** 10.1038/ncomms13216

**Published:** 2016-10-18

**Authors:** Qiufang Gong, Yu Wang, Qi Hu, Jigang Zhou, Renfei Feng, Paul N. Duchesne, Peng Zhang, Fengjiao Chen, Na Han, Yafei Li, Chuanhong Jin, Yanguang Li, Shuit-Tong Lee

**Affiliations:** 1 Institute of Functional Nano & Soft Materials (FUNSOM), Jiangsu Key Laboratory for Carbon-Based Functional Materials and Devices, Soochow University, Suzhou 215123, China; 2 College of Chemistry and Materials Science, Nanjing Normal University, Nanjing 210023, China; 3 State Key Laboratory of Silicon Materials, School of Materials Science and Engineering, Zhejiang University, Hangzhou, Zhejiang 310027, China; 4 Canadian Light Source Inc., Saskatoon, Saskatchewan, Canada S7N 0X4; 5 Department of Chemistry, Dalhousie University, Halifax, Canada NS B3H 4R2

## Abstract

Earlier research has been primarily focused on WC as one of the most promising earth-abundant electrocatalysts for hydrogen evolution reaction (HER), whereas the other compound in this carbide family—W_2_C—has received far less attention. Our theoretical calculations suggest that such a focus is misplaced and W_2_C is potentially more HER-active than WC. Nevertheless, the preparation of phase pure and sintering-free W_2_C nanostructures represents a formidable challenge. Here we develop an improved carburization method and successfully prepare ultrasmall and phase-pure W_2_C nanoparticles. When evaluated for HER electrocatalysis, W_2_C nanoparticles exhibit a small onset overpotential of 50 mV, a Tafel slope of 45 mV dec^−1^ and outstanding long-term cycling stability, which are dramatically improved over all existing WC-based materials. In addition, the integration of W_2_C nanoparticles with p-type Si nanowires enables highly active and sustainable solar-driven hydrogen production. Our results highlight the great potential of this traditionally non-popular material in HER electrocatalysis.

## Abstract (web summary)

Tungsten carbide has yet to live up to its long-believed potential as a replacement for precious metal electrocatalysts. Here, Li and co-workers demonstrate that ditungsten carbide in the form of ultrasmall, phase-pure nanoparticles is a better candidate for the hydrogen evolution reaction.

## Graphical abstract

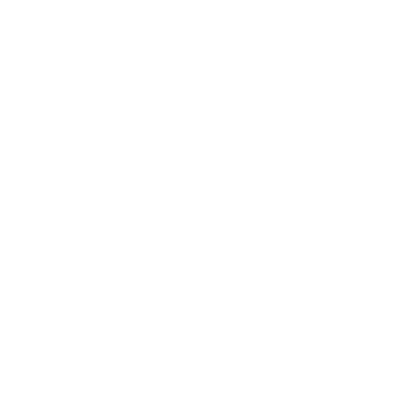

Splitting water molecules to gaseous hydrogen and oxygen using renewable resources such as solar energy represents one of the biggest challenges facing humanity in the modern age^1^,^2^,^3^,^4^. Among several obstacles to its practical realization at a large scale is the development of active and durable catalyst materials for hydrogen evolution reaction (HER) at the cathode and oxygen evolution reaction (OER) at the anode^4^,^5^,^6^,^7^. Even though OER remains the bottleneck of water splitting, HER is no less challenging. Platinum group metals are the most active HER electrocatalysts, yet all of them suffer from low abundance and high cost^4^,^6^,^7^. The search for non-precious metal based alternatives has never been more demanding than it is today. Despite the recent exciting advances of many transition metal chalcogenide, nitride or phosphide-based HER electrocatalysts, there are few candidates available at present that can simultaneously meet the activity and stability requirements^4^,^5^,^6^,^7^.

Early transition metal carbides have attracted wide interest owing to their low cost, high chemical and electrochemical stability, and unique catalytic activities towards a range of important reactions^8^,^9^,^10^,^11^. Theoretical calculations have demonstrated that the presence of carbon interstitial atoms in the lattice of early transition metals can afford them with higher d-band electronic density of states (DOS) at the Fermi level and hence characteristics resembling those of platinum^11^,^12^. In particular, tungsten carbide (WC)-based materials have been long advocated and investigated as potential replacements of platinum for HER electrocatalysis ever since the first report by Trasatti more than half a century ago^13^. Unfortunately, these materials have yet to be capable of replacing precious metal catalysts due to their poor activities: very few have onset overpotentials within 100 mV (refs 7, 10, 14, 15). The challenges are multifold^7^,^10^,^15^,^16^. First, these carbides have been conventionally prepared using high-temperature (>700 °C) reduction of metal precursors with gaseous carbon precursors (such as CH_4_, C_2_H_6_ or CO), which usually induces uncontrollable particle sintering, resulting in materials with extremely low surface areas. Second, the introduction of excessive gaseous carbon precursors may also risk extensive coking of catalyst surfaces, seriously deteriorating its catalytic performance. Third and most importantly, there are very few existing methods to selectively engineer different phases of carbides for maximal HER activity. For decades, research on tungsten carbides has been exclusively focused on WC. The other carbide compound—W_2_C has received far less attention. It is mainly due to the lack of a reliable synthetic method for this carbon-deficient material. Among the few reports available up to date, W_2_C is usually obtained as a by-product of WC^17^,^18^. This is not surprising since the formation of W_2_C is not thermodynamically favoured below 1,250 °C according to the W–C phase diagram^19^. Despite its unpopularity among material scientists and electrochemists, our theoretical calculations predict that W_2_C is a significantly more active HER catalyst than WC with a much less negative Gibbs free energy of hydrogen adsorption (Δ*G*_H_) and higher electronic DOS at the Fermi level. As a result, the preparation of nanosized, coking-free and phase-pure W_2_C particles and assessment of their potential in HER electrocatalysis are a daunting but highly rewarding task. Yet, nobody has been able to accomplish this up to now.

In this study, we advance a solution to this challenge via the carburization reaction between non-volatile, crystalline solid carbon precursor and pre-formed WO_*x*_ nanoparticles at high temperature and low pressure. Multi-walled carbon nanotubes (MWNTs) are selected as such a solid carbon precursor. Due to their chemical inertness, the diffusion rate of carbon atoms from MWNTs to tungsten lattice is slowed down, which not only leads to suppressed sintering of carbide nanoparticles, but also avoids excessive carbon deposition on their surfaces. In this way, we are able to achieve the selective synthesis of ultrasmall and phase-pure W_2_C particles with excellent activity and durability for both electrocatalytic and photoelectrochemical hydrogen evolution.

## Results

### Theoretical calculations

**Figure 1:**
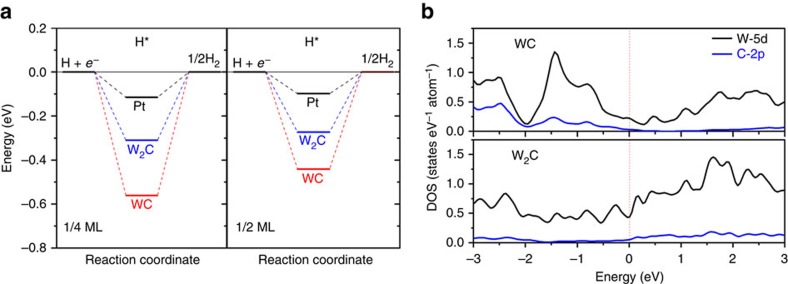
DFT computations on the HER activity and electronic structure of W_2_C. (**a**) Free-energy diagrams for HER on the (0001) surfaces of W_2_C and WC, as well as Pt (111) surface at both low (1/4 ML_H_) and high (1/2 ML_H_) hydrogen adsorption coverage. (**b**) Calculated partial density of states of W and C atoms in W_2_C and WC. The red dashed line denotes the position of the Fermi level.

We start with the first-principles density functional theory (DFT) calculations to unveil the greater potential of W_2_C over WC for HER electrocatalysis. Δ*G*_H_ has been advocated as a useful descriptor in the selection of proper HER electrocatalysts^20^,^21^,^22^,^23^,^24^. According to the Sabatier principle, the optimal electrocatalysts should have Δ*G*_H_ neither too negative nor too positive (Δ*G*_H_ ≈0 eV) as we have seen for the case of platinum^22^,^23^. Here, we first computed and compared the Δ*G*_H_ on the (0001) surface of hexagonal WC and W_2_C, alongside with the (111) surface of cubic Pt as a reference. The three-state diagram of HER free-energy process in Fig. 1a shows that at the low hydrogen adsorption coverage of 1/4 monolayer (ML_H_), the Δ*G*_H_ on WC is −0.56 eV, in good agreement with the result reported by Peterson *et al*. and suggesting that hydrogen intermediates adsorb onto WC surfaces more strongly than ideal^25^. Under the same condition, the Δ*G*_H_ on W_2_C is only −0.31 eV. This value represents a significant improvement over WC, even though it is still slightly more negative than the Pt reference (which has a calculated Δ*G*_H_ of −0.11 eV in our study, close to the result reported by Nørskov *et al*.^21^) and indicates that HER on W_2_C is still limited by the hydrogen release step. At an increased hydrogen adsorption coverage of 1/2 ML_H_, Δ*G*_H_ on WC and W_2_C shifts positively, and reaches −0.44 and −0.27 eV, respectively (Fig. 1a). It is therefore conclusive that HER is more favoured at high hydrogen coverage for both W_2_C and WC, nevertheless the former remains far superior to the latter.

Other than its less negative Δ*G*_H_, the advantage of W_2_C over WC as the HER electrocatalyst is also manifested from the comparison of their electronic structures. Figure 1b illustrates the partial density of states of individual W and C atoms in WC and W_2_C. WC has long been suggested as platinum-like because of its modified d-band electron structure with a greater density of states (DOS) near the Fermi level upon the incorporation of carbon interstitial atoms^11^,^12^. This is again confirmed by our study here showing the considerable W 5d contribution at the Fermi level. Interestingly, we find that electronic DOS of W_2_C near the Fermi level is even larger than that of WC. The higher W content in W_2_C enhances its metallicity, and results in a higher carrier density, which would greatly benefit the charge transfer process during electrochemical reactions^26^. Furthermore, we calculate that the d-band center of W_2_C (0001) (−1.74 eV) is close to that of Pt (111) (−1.92 eV), but much lower than that of WC (0001) (−0.93 eV; Supplementary Fig. 1). According to the d-band theory, a lower d-band center results in weaker bonding between the catalyst and the adsorbate. This result rationalizes the more favourable Δ*G*_H_ on W_2_C than on WC, and suggests that the former is more Pt-like than the latter.

**Figure 2:**
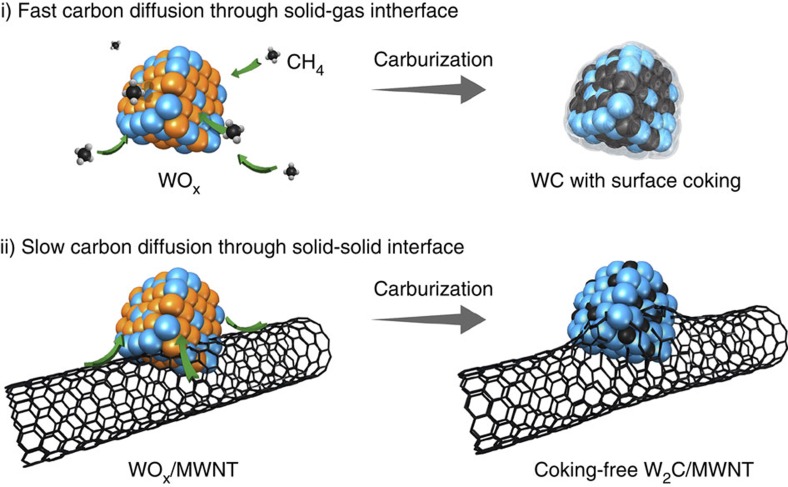
Schematic illustration showing the influence of different carbon precursors. Gaseous carbon precursor (for example, CH_4_) and non-volatile solid carbon precursor (for example, CNT) may have different impacts on the chemical composition and microstructure of the carburization product.

The above DFT calculations have unambiguously established that W_2_C is a potentially more active HER electrocatalyst than WC because of its more favourable Δ*G*_H_ and higher electronic DOS. However, the preparation of nanosized W_2_C is challenging, and so far not successful. The W–C phase diagram suggests that the formation of W_2_C is not thermodynamically favoured below 1,250 °C (ref. 19). Moreover, the synthesis has to be carried out in a carbon-deficient environment to avoid the undesired formation of WC (see Supplementary Table 1 for the summarized synthetic methods of W_2_C)^27^,^28^,^29^,^30^,^31^. Most conventional approaches (such as those using gaseous carbon precursors) are unable to yield W_2_C as the main product since the relative amount of carbon precursor to tungsten precursor is often out of control, and the carbon diffusion through the solid-gas interface into the tungsten lattice usually proceeds too fast to allow for the product phase to be carefully engineered (Fig. 2a)^10^,^15^. Here, we reason that using a non-volatile, crystalline solid carbon precursor such as carbon nanotubes may present a unique solution to this problem. Under high temperatures, the chemical reactivity of non-volatile solid carbon precursors is considerably lower. They would render the carbon diffusion through the solid-solid interface much slower, opening up the possibility to tailor the product phase via selecting proper reaction conditions (Fig. 2b).

### Synthesis of W_2_C nanoparticles

Guided by this design principle, we report here a two-step approach to prepare ultrasmall and phase-pure W_2_C particles supported on MWNTs (see the Methods section for details). In the first step, ultrasmall WO_*x*_ nanoparticles were grown on pre-oxidized MWNTs via the controlled hydrolysis of WCl_6_ in a mixed solvent of ethanol and deionized water (Supplementary Fig. 2). This was mediated through the strong interaction between metal precursors and rich oxygen functionalities on MWNTs^32^,^33^,^34^. Subsequently, the hybrid was subjected to carburization at simultaneously high temperature and low pressure, during which carbon atoms from the MWNT support are properly activated, diffuse into WO_*x*_ lattices, and selectively reduce them to W_2_C (Fig. 2b). The optimal conditions to afford small-sized and phase-pure product were found to be 900 °C and 320 Pa.

**Figure 3:**
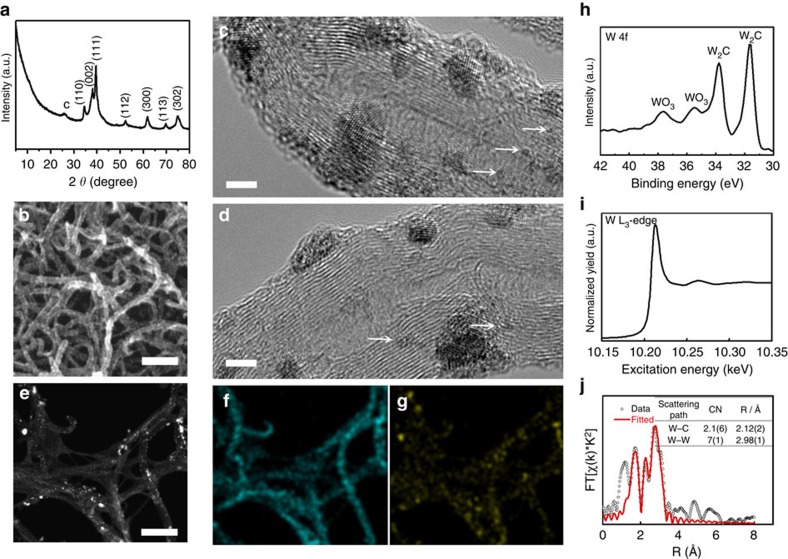
Microscopic and spectroscopic characterizations of W_2_C nanoparticles supported on MWNTs. (**a**) X-ray diffraction pattern, (**b**) SEM image and (**c**,**d**) TEM images of W_2_C/MWNT. (**e**) HAADF image and (**f**,**g**) corresponding C and W EDS element mapping of W_2_C/MWNT. (**h**) W 4f XPS spectrum, (**i**) W L_3_-edge XANES spectrum and (**j**) Fourier transform EXAFS spectrum and associated fitting curve of W_2_C/MWNT at the W L_3_-edge. The inset in **j** shows the fitting results. Scale bar, 100 nm (**b**,**e**); 3 nm (**c**,**d**).

We first examined the chemical composition and microstructure of the final product using X-ray powder diffraction (XRD) and electron microscopy as summarized in Fig. 3a. From X-ray diffraction, except for the weak signal at 25.7^o^ from MWNTs, all the diffraction peaks are assignable to W_2_C with no sign of the formation of WC or other species. Scanning electron microscopy (SEM) shows that after the high-temperature carburization, MWNTs retain the one-dimensional morphology and are uniformly decorated with small W_2_C nanoparticles on the surfaces (Fig. 3b). Unlike conventional carbide-based materials, no further aggregation of W_2_C nanoparticles or their detachment from the support is evidenced here owing to their strong physical immobilization on the conductive scaffold. Fig. 3c,d are the representative high-resolution transmission electron microscopy (TEM) images of the final product. Dark contrast W_2_C nanoparticles can be readily distinguished from the MWNT background. Most of them have a fine size between 2 and 5 nm with clear lattice fringes. In addition, we also identify some smaller clusters having sizes in the sub-nanometer range such as those marked by arrows. The distribution of W_2_C nanoparticles on MWNTs over a large area was garnered using high-angle annular dark field (HAADF) scanning transmission electron microscopy (STEM) imaging, and energy dispersive spectroscopy (EDS) elemental mapping under STEM mode. HAADF imaging has a significantly higher sensitivity towards heavy elements^35^. Bright spots in Fig. 3e reveal the spatial dispersion of W species along MWNTs. Most of them are no larger than a few pixels, thereby attesting to the ultrasmall size of W_2_C nanoparticles. The EDS signal of W corresponds well with the HAADF image (Fig. 3f). Its high spatial correlation with the C signal (Fig. 3g) also supports the uniform distribution of W_2_C nanoparticles on MWNTs. The weight percentage of W_2_C in the composite is analysed to be ∼69 wt% by thermogravimetric analysis (Supplementary Fig. 3).

To gain insight on the chemical environment and bonding configuration of W_2_C nanoparticles, multiple spectroscopic characterizations were performed on the carburized product. Its W 4f X-ray photoelectron spectroscopy (XPS) spectrum is featured with two pronounced peaks centered at 31.7 eV and 33.8 eV, typical to tungsten carbides in general (Fig. 3h)^36^,^37^. Peaks at the higher binding energy of 35.5 and 37.6 eV are resulted from the inevitable surface oxidation of W_2_C nanoparticles upon exposure to air^36^,^38^. Figure 3i,j depict the W L_3_-edge XANES spectrum and its corresponding Fourier-transformed extended X-ray absorption fine structure (EXAFS) spectrum of W_2_C. From the theoretical fitting of the latter, we derive an average W–C bond length of 2.12 Å and W–W bond length of 2.98 Å (see the inserted table in Fig. 3j). These values are more consistent with the crystal structure of hexagonal W_2_C than WC, and again corroborate the formation of W_2_C instead of WC. Most importantly, the coordination numbers (CN) of W–C and W–W are fit to be 2.1±0.6 and 7±1, respectively. Both are markedly smaller compared with the expected values for bulk materials (CN=6 for W-C and CN=12 for W–W). This under-coordination can be rationalized by the ultrasmall size and defective nature of the resultant W_2_C nanoparticles, in good agreement with above TEM results.

The above microscopic and spectroscopic studies establish that using MWNTs as the non-volatile solid carbon precursor, we have accomplished the selective carburization to form ultrasmall and phase-pure W_2_C nanoparticles. It is worth highlighting that MWNTs play a pivotal role in our control over both the size and phase of the carbide nanoparticles. Among many previous attempts to mitigate particle sintering, Romón–Leshkov and coworkers developed a removable ceramic coating method by encapsulating WO_*x*_ within SiO_2_ nanospheres as a hard template to physically prevent their sintering during the high-temperature carburizing^15^. Here we believe that similar physical confinement can also be in effect via the strong interaction between W precursors and their supporting materials. When firmly immobilized on the conductive MWNT scaffold, the further aggregation or overgrowth of W_2_C nanoparticles is fully suppressed. On the other hand, the slow carbon diffusion from MWNTs to WO_x_ through the solid–solid interface creates an overall carbon-deficient environment, which is responsible for the favorable formation of W_2_C over the more common WC phase (Fig. 2b). It is in stark contrast to conventional carbide preparation methods using gaseous carbon precursors (such as CH_4_, C_2_H_6_ or CO), where the carbon diffusion through the solid–gas interface usually proceeds too fast to be controlled (Fig. 2a)^10^,^15^,^16^,^18^,^39^. Indeed, our control experiment shows that when CH_4_ is fed as the gaseous carbon precursor under otherwise identical conditions, the final product is exclusively comprised of WC with a similar size distribution (Supplementary Fig. 4).

We find that carburization conditions also have a large influence on the phase and size of the final product. When the carburization temperature is raised from 900 °C to 1,000–1,100 °C, WC phase emerges and grows in percentage based on X-ray diffraction analysis as the result of the accelerated carbon diffusion (Supplementary Fig. 5)^14^,^28^. When the temperature is maintained at 900 °C, annealing in vacuum (5 Pa) or atmospheric pressure results in predominantly metallic W or WO_2_ phase, respectively, with drastically different morphologies (Supplementary Fig. 6). Moreover, cubic or hexagonal WN can be prepared when the carrier gas is switched from N_2_ to NH_3_ (Supplementary Fig. 7).

### Electrocatalytic HER performance of W_2_C

**Figure 4:**
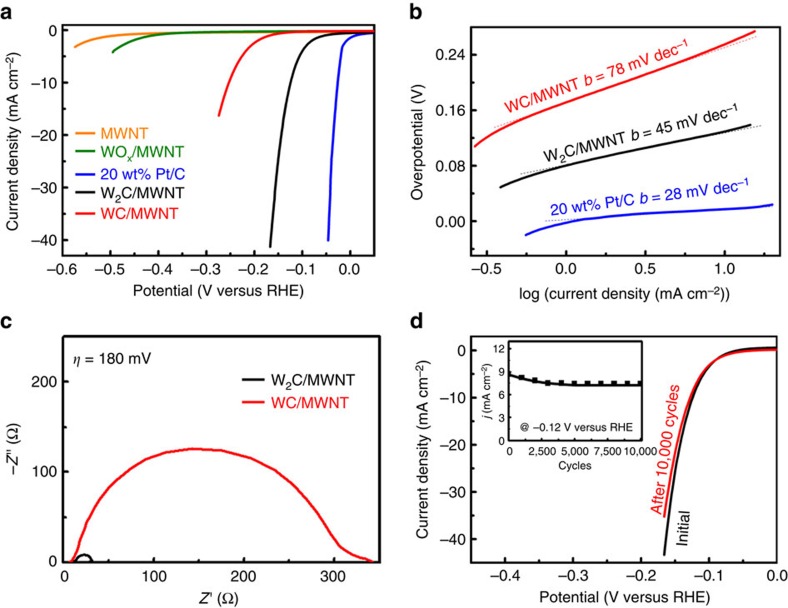
Electrochemical measurements of W_2_C/MWNT for HER in 0.5 M H_2_SO_4_. (**a**) Polarization curve of W_2_C/MWNT in comparison with 20 wt% Pt/C, WC/MWNT, WO_x_/MWNT or MWNTs alone. (**b**) Tafel plot of W_2_C/MWNT in comparison with 20 wt% Pt/C and WC/MWNT. (**c**) Electrochemical impedance spectrum of W_2_C/MWNT at *η*=180 mV in comparison with WC/MWNT. (**d**) Polarization curve of W_2_C/MWNT before and after 10,000 potential cycles. The inset shows the change of HER cathodic current density at −0.12 V versus RHE with respect to the number of potential cycles.

The electrocatalytic performance of W_2_C nanoparticles for HER was assessed by using a standard three-electrode setup in 0.5 M H_2_SO_4_ (see details in Methods section). HER polarization curves of different electrocatalysts were collected and compared alongside with the commercial 20 wt% Pt/C benchmark. As shown in Fig. 4a, MWNTs alone or WO_*x*_/MWNT from the first step has negligible HER activity. By contrast, W_2_C/MWNT exhibits an impressive HER activity with a small onset overpotential of ∼50 mV and achieving a geometric current density of 10 mA cm^−2^ at η=123 mV versus reversible hydrogen electrode (RHE). Such an activity excels most previous carbide-based (WC and Mo_2_C) HER electrocatalysts to our best knowledge, and is among the best non-precious metal based electrocatalysts (Supplementary Table 2)^7^,^10^,^15^,^28^,^40^. Tafel analysis yields a small slope of 45 mV dec^−1^ for W_2_C/MWNT (Fig. 4b), again representing the smallest value ever reported, and indicative of possible Volmer–Heyrovsky pathway with the electrochemical desorption reaction as the rate determining step^41^. Its fast HER reaction kinetics is also reflected by a small charge transfer resistance of 20 Ω at *η*=180 mV from electrochemical impedance spectroscopy analysis (Fig. 4c). Interestingly, we note that WC/MWNT prepared using CH_4_ as the feed gas in our experiment has much inferior performance (Supplementary Fig. 4). It delivers *j*=10 mA cm^−2^ at *η*=250 mV; its charge transfer resistance at *η*=180 mV is 15 times larger than W_2_C/MWNT—a performance consistent with the best WC-based HER electrocatalysts (such as the one reported by Romón–Leshkov using the removable ceramic coating method^15^). Given the largely similar size distribution of WC and W_2_C nanoparticles here, we believe that the striking difference in their HER activities clearly underscores the advantageous electronic structure of W_2_C over WC as elucidated by the above DFT calculations.

Meanwhile, it is worth emphasizing that the ultrasmall size of W_2_C is also an indispensable structural factor to achieve its superior HER activity. Since electrocatalysis is a surface-limiting process, a smaller particle size results in larger surface areas and exposes more catalytically active sites for HER. To estimate its electrochemical surface area (ECSA), W_2_C is electrochemically oxidized in an anodic sweep (Supplementary Fig. 8). From the charge integrated over the oxidation envelope, we estimate ∼60% of W_2_C is electrochemically accessible. This value is very impressive and consistent with the ultrasmall size of W_2_C particles. By contrast, non-structured W_2_C is significantly less active. For example, W_2_C thin film prepared from the magnetron sputtering only has a mediocre HER activity with *j*=1 mA cm^−2^ achieved at *η*∼200 mV (ref. 30).

HER activities of a range of control samples are summarized in the Supplementary Information. We have systematically explored the influence of starting precursor ratio (Supplementary Fig. 9), carburization temperature (Supplementary Fig. 5), pressure (Supplementary Fig. 6), and carrier gas (Supplementary Fig. 4 and 7), and concluded that the present conditions yield the most active HER electrocatalyst.

Furthermore, W_2_C/MWNT has satisfactory long-term stability. To demonstrate this, the catalyst was continuously cycled up to 10,000 cycles. Its polarization curve was periodically collected every 1,000 cycles, and cathodic current density at *η*=120 mV extracted and plotted against the cycle number as depicted in the insert of Fig. 4d. We can see that despite a slight initial activity loss, the cathodic current density levels off after 4,000 cycles, and remains constant at ∼7.4 mA cm^−2^. When the initial and final polarization curves are compared in Fig. 4d, this activity loss translates to <10 mV increment in overpotential. The outstanding cycling stability of W_2_C/MWNT attests to its great structural and electrochemical robustness. Postmortem X-ray diffraction and XPS analysis of W_2_C/MWNT after HER reveal that its crystallinity is retained while its surface becomes slightly more oxidized (Supplementary Fig. 10).

### PEC hydrogen generation on W_2_C decorated Si nanowires

**Figure 5:**
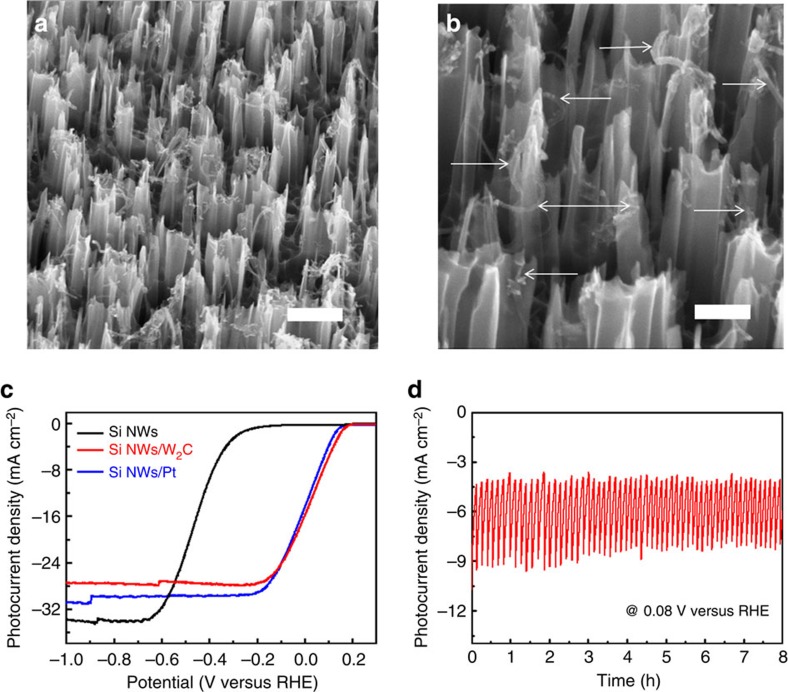
Integration of W_2_C/MWNT with p-type Si nanowires for PEC hydrogen generation in 0.5 M H_2_SO_4_. (**a**,**b**) SEM images of the photocathode loaded with W_2_C/MWNT catalyst as marked by arrows. Scale bar, 400 nm (**a**) and 200 nm (**b**). (**c**) Polarization curve of Si nanowire photocathode loaded with W_2_C/MWNT under one sun illumination in comparison with the same photocathode loaded with 20 wt% Pt/C or Si nanowire photocathode alone. (**d**) Amperometric *i−t* curve at a constant potential of 0.08 V versus RHE under one sun illumination.

We integrated W_2_C/MWNT with p-type Si nanowires and studied their performance for photoelectrochemical (PEC) hydrogen evolution. P-type Si nanowires having an average length of 500 nm were prepared from the Ag-assisted etching of Si wafer (see details in Methods)^42^,^43^. W_2_C/MWNT was directly dropcast onto these nanowires. Figure 5a shows a tilted SEM image of the photocathode. These vertically aligned Si nanowires have adequate open space among them, and are uniformly infiltrated with W_2_C/MWNT catalysts over a large area. Close examination reveals that many W_2_C/MWNT fibers coil around individual Si nanowires or bridge neighbouring Si nanowires (marked by arrows in Fig. 5b). Even though Si nanowires may not be in direct physical contact with W_2_C nanoparticles, they are electrically connected through MWNTs.

The PEC polarization curves of Si nanowire photocathodes with or without catalyst under 1 sun illumination are presented and compared in Fig. 5c. Si nanowires alone have a poor PEC activity with no appreciable photocurrent density to the positive of −0.2 V versus RHE. After integration of Si nanowires with W_2_C/MWNT, their onset potential improves significantly to 0.2 V, and a photocurrent density of 16 mA cm^−2^ is reached at 0 V. Both metrics are well comparable to the photocathode loaded with 20 wt% Pt/C, and superior to previous p-type Si nanowire electrodes integrated with other earth-abundant electrocatalysts, such as Mo_3_S_4_, Ni–Mo alloy or Mo_2_C (Supplementary Table 3)^44^,^45^,^46^,^47^. Note that the slightly lower saturation photocurrent density of catalyst-loaded Si nanowires (∼30 mA cm^−2^) compared with bare Si nanowires (∼34 mA cm^−2^) probably suggests that a fraction of the incoming light is blocked by the catalyst powder. Moreover, the operation durability of Si/W_2_C/MWNT was investigated by galvanostatic (*i*∼*t*) experiment at 0.08 V up to 8 h (Fig. 5d). Even though the photocurrent density fluctuates due to the periodic accumulation and release of H_2_ bubbles from the photocathode surface, there is no sign of PEC activity decay over the course of experiment. All these results would not be possible if it was not for the great HER activity and durability of our W_2_C/MWNT catalyst. The physical integration of Si nanowires and W_2_C/MWNT is also strong enough to survive the vigorous bubbling on the electrode surface.

## Discussion

In conclusion, built on our improved understanding of the pitfalls associated with conventional preparation methods and the great potential of W_2_C predicted by theoretical calculations, we demonstrated the selective synthesis of ultrasmall and phase-pure W_2_C particles using non-volatile crystalline solid carbon precursor (that is, MWNTs), and achieved efficient and sustainable electrocatalytic and photoelectrochemical hydrogen evolution. The slow carbon diffusion from MWNT support to WO_*x*_ nanoparticles through the solid–solid interface created an overall carbon-deficient environment, and led to the favorable formation of W_2_C over the more common WC. It also prevented the extensive coking and sintering of the resultant carbide nanoparticles. As a result, W_2_C/MWNT exhibited impressive electrocatalytic HER performance, featuring a small onset overpotential of ∼50 mV, a cathodic current density of 10 mA cm^−2^ at *η*=123 mV, and long-term cycling stability. W_2_C/MWNT is far superior to all existing WC-based HER electrocatalysts. In addition, W_2_C/MWNT could be readily integrated with p-type Si nanowires to enable solar-driven hydrogen production, and achieve highly competitive performance to that of the photocathode decorated with Pt. We also expect our ultrasmall W_2_C nanoparticles to be more active than WC towards a range of other catalytic or electrocatalytic reactions.

## Methods

### Materials synthesis

To prepare W_2_C/MWNT, MWNTs were first oxidized by modified Hummers method following a previous report^48^. In brief, 1 g of MWNTs were oxidized by 200 mg of NaNO_3_ and 1 g of KMnO_4_ in 23 ml of concentrated H_2_SO_4_ at 40 °C for 30 min. Subsequently, 16 mg of pre-oxidized MWNTs and 128 mg of anhydrous WCl_6_ were dispersed in 40 ml of ethanol and 4 ml of water. The temperature of the solution was then raised to and maintained at 80 °C overnight in an oil bath under vigorous magnetic stirring. After cooling back to room temperature, the solid product was collected by centrifugation, repeatedly washed with absolute ethanol and water, and lyophilized. To carry out the carburization reaction, the as-prepared solid powder was placed in the center of a tube furnace. The system was evacuated to 320 Pa at a continuous Ar flow rate of 100 sccm. The specimen was heated to 900 °C at a ramping rate of 10 °C min^−1^, and maintained at this temperature for 1 h.

### Structure characterizations

Powder X-ray diffraction was performed on a PANalytical X-ray diffractometer at a scan rate of 0.05° s^−1^. XPS were collected on an Ultra-DLD XPS Spectrometer. Scanning electron microscopy (s.e.m.) images were taken on a Supera 55 Zeiss scanning electron microscope. TEM was done with a FEI F20 microscope, and the HAADF-STEM and STEM-EDS mapping were carried out on a FEI Titan G^2^ 80–200 microscope. Both of them were operated at an acceleration voltage of 200 kV. W L3 edge XANES spectra were collected on the VESPERS (Very Sensitive Elemental and Structural Probe Employing Radiation from a Synchrotron) beamline at the Canadian Light Source. The energy scan was achieved by using a double crystal Si(111) monochromator. The emitted X-ray fluorescence spectrum was recorded by a 4-element Vortex silicon drift detector, which is positioned 50 mm away from the sample at 45 degree angle to the sample and 90 degrees to the incoming beam within the horizontal plane. Subsequently, the XANES spectrum was background-subtracted using low-order polynomials and normalized based on its post-absorption edge intensity. EXAFS data fitting was performed using WinXAS software in conjunction with scattering paths generated by FEFF8 (refs 49, 50). Absorption edge-shift (Δ*E*_0_) values were correlated between the two scattering paths, and all other parameters were allowed to vary freely. Both W–C and W–W bond lengths were obtained from a series of tungsten carbide structures and compared to those obtained experimentally from the fitted EXAFS spectra.

### Electrochemical measurements

To prepare the catalyst ink, 1 mg of active materials, 0.5 mg of Ketjenblack carbon and 5 μl of Nafion solution (5 wt%) were dispersed in a mixture of 250 μl of H_2_O and ethanol (ν/ν, 1/1) with the assistance of ultrasonication for at least 30 min. Then 10 μl of the ink was dropcast onto a glassy carbon electrode of 3 mm in diameter (catalyst loading ∼0.56 mg cm^−2^). Electrochemical measurements were performed in a standard three-electrode set-up in 0.5 M H_2_SO_4_ solution, using a saturated calomel electrode (SCE), the catalyst loaded glassy carbon electrode, and a graphite rod as the reference, working and counter electrode, respectively. Before each measurement, the SCE reference electrode was carefully calibrated against a commercial RHE from ASL Japan. Linear sweep voltammetry (LSV) was carried out at a scan rate of 10 mV s^−1^. Electrochemical impedance measurements were carried out at different overpotentials in the frequency range of 100,000–0.05 Hz.

### Fabrication of photocathode and PEC measurements

Si nanowire arrays were fabricated via the metal assisted chemical etching of Si wafers. In brief, a (100)-oriented Si wafer (p-type, 0.2–0.8 Ω cm^−1^) was first degreased by ultrasonication in ethanol and acetone sequentially, each for 15 min. It was then treated in a boiling piranha solution (H_2_O_2_:H_2_SO_4_=1:4 v/v) for 30 min. Thus cleaned Si wafer was etched in an aqueous etchant containing 20 mM AgNO_3_ and 5 M HF for 15 min, subsequently treated in 5% HNO_3_ solution for 30 min to remove surface Ag residue. Resultant Si nanowire arrays were rinsed with a copious amount of water, and finally dried at 80 °C for further use. To integrate Si nanowire arrays with W_2_C nanoparticles, Si nanowires were etched in 0.5 M HF for 15 s and were blow-dried under N_2_ purge. Ten microlitre of 4 mg ml^−1^ W_2_C/MWNT in ethanol dispersion was uniformly spin-coated onto a 15 mm × 15 mm Si nanowire electrode at 2,500 r.p.m., and naturally dried in air. Ohmic contact to the backside of electrode was made by scratching with Ga–In eutectic and affixing a Cu wire to it. PEC experiments were carried out in a custom-built square cell with a circular 0.38 cm^2^ aperture, which was sealed by the working electrode. Light was provided by a 300 W Xe arc lamp from Newport Corporation fitted with an AM 1.5 G filtre. Its power density was adjusted to 100 mW cm^−2^, and calibrated with an optical power meter equipped with a thermopile detector. LSV curves were collected at a scan rate of 10 mV s^−1^.

### Theoretical calculations

DFT calculations were performed using the plane-wave technique with exchange-correlation interactions modeled by the revised Perdew–Burke–Ernzerhof generalized gradient approximation functional^51^, as implemented in the Vienna *ab Initio* Simulation package^52^,^53^. The ion–electron interaction was treated within the projector-augmented plane wave pseudopotentials^54^,^55^. A plane-wave cutoff energy of 420 eV was used in all computations. The electronic structure calculations were employed with a Fermi-level smearing of 0.1 eV for all surface calculations and 0.01 eV for gas-phase species. The Brillouin zone was sampled with 12 × 12 × 12 for bulk calculations and 6 × 6 × 1 k-points for surfaces calculations. The convergence of energy and forces were set to be 1 × 10^−5^ eV and 0.02 eV Å^−1^, respectively. For all surfaces, a 2 × 2 supercell was used and a vacuum region of 15 Å was set along the *z* direction to avoid the interaction between periodic images (Supplementary Fig. 11). The Pt (111) surface was modeled with four atomic layers where only the top layer and adsorbate were allowed to fully relax. The WC(0001) and W_2_C (0001) planes were, respectively, constructed with ten and nine atomic layers respectively where the top two layers and adsorbate were allowed to fully relax.

The free energy of adsorbed H (Δ*G*_H_) on different surfaces is expressed as:


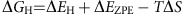


where Δ*E*_H_ is the hydrogen adsorption energy, Δ*E*_ZPE_ and Δ*S* are the zero point energy difference and the entropy difference between the adsorbed state and the gas phase, respectively, and *T* is the system temperature (298.15 K, in this work). For each system, its Δ*E*_H_ was directly determined from the DFT total energies, and its *E*_ZPE_ was computed by summing vibrational frequencies over all normal modes ν (*E*_ZPE_=1/2Σ*ħν*). After exploring possible H adsorption sites, we reported the lowest energy binding location as shown in Supplementary Fig. 11. The free energy of proton and electron (H^+^+*e*^−^) at standard conditions was taken as 1/2*G*_H2_.

### Data availability

The data that support the findings of this study are available from the corresponding author upon reasonable request.

## Additional information

**How to cite this article:** Gong, Q. *et al*. Ultrasmall and phase-pure W_2_C nanoparticles for efficient electrocatalytic and photoelectrochemical hydrogen evolution. *Nat. Commun.* **7,** 13216 doi: 10.1038/ncomms13216 (2016).

## Supplementary Material

Supplementary Information Supplementary Figures 1 - 11 and Supplementary Tables 1 - 3
