# Supplementary material for: Ultrasmall and phase-pure W2C nanoparticles for efficient electrocatalytic and photoelectrochemical hydrogen evolution
Source: Nat Commun. 2016 Oct 18;7:13216. doi: 10.1038/ncomms13216 (PMC5071847; doi:10.1038/ncomms13216)
Supplement: Supplementary Information — Supplementary Figures 1 - 11 and Supplementary Tables 1 - 3 [file ncomms13216-s1.pdf]

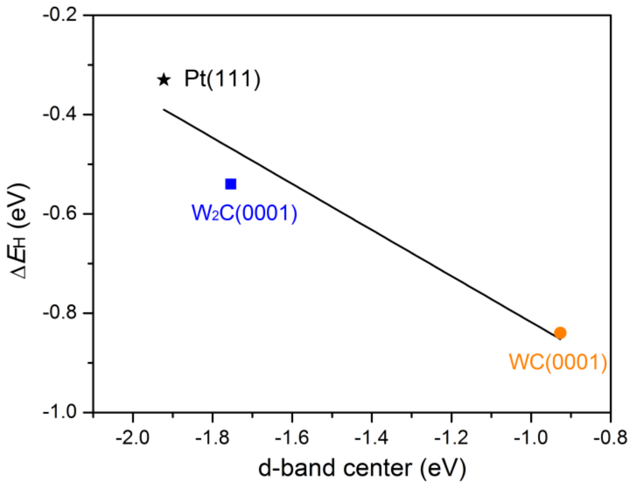

Supplementary Figure 1. Correlation of hydrogen adsorption energy (ΔE_H) at 0.25 ML_H with the d-band center over Pt (111), WC (0001), and W_2C (0001).

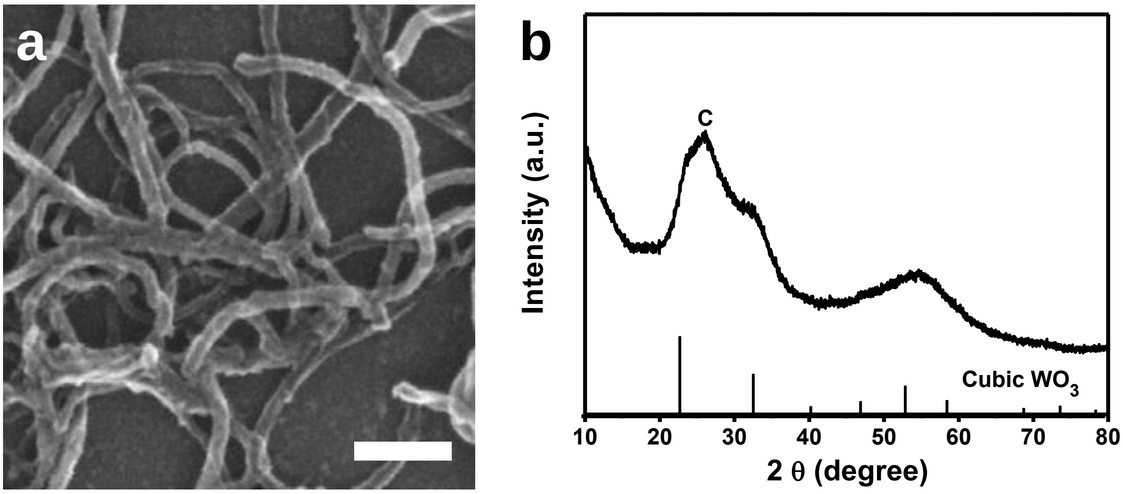

Supplementary Figure 2. (a) SEM image (scale bar, 100 nm) and (b) XRD pattern of WO_x/MWNT from the first step.

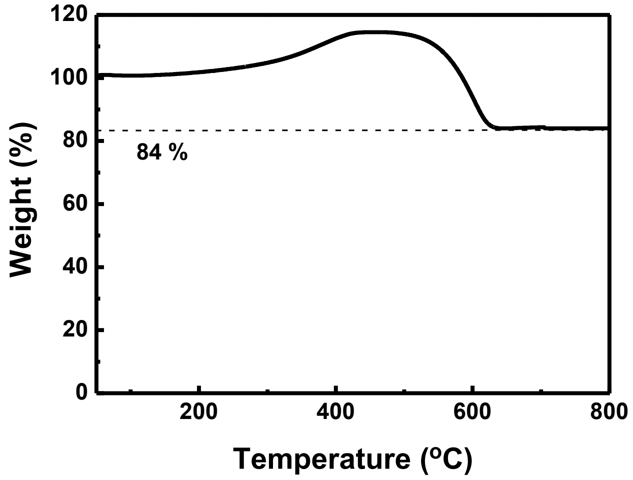

Supplementary Figure 3. TGA curve of W_2C/MWNT in air from 50 to 800°C at a heating rate of 20 ^{o}C min^{-1}.

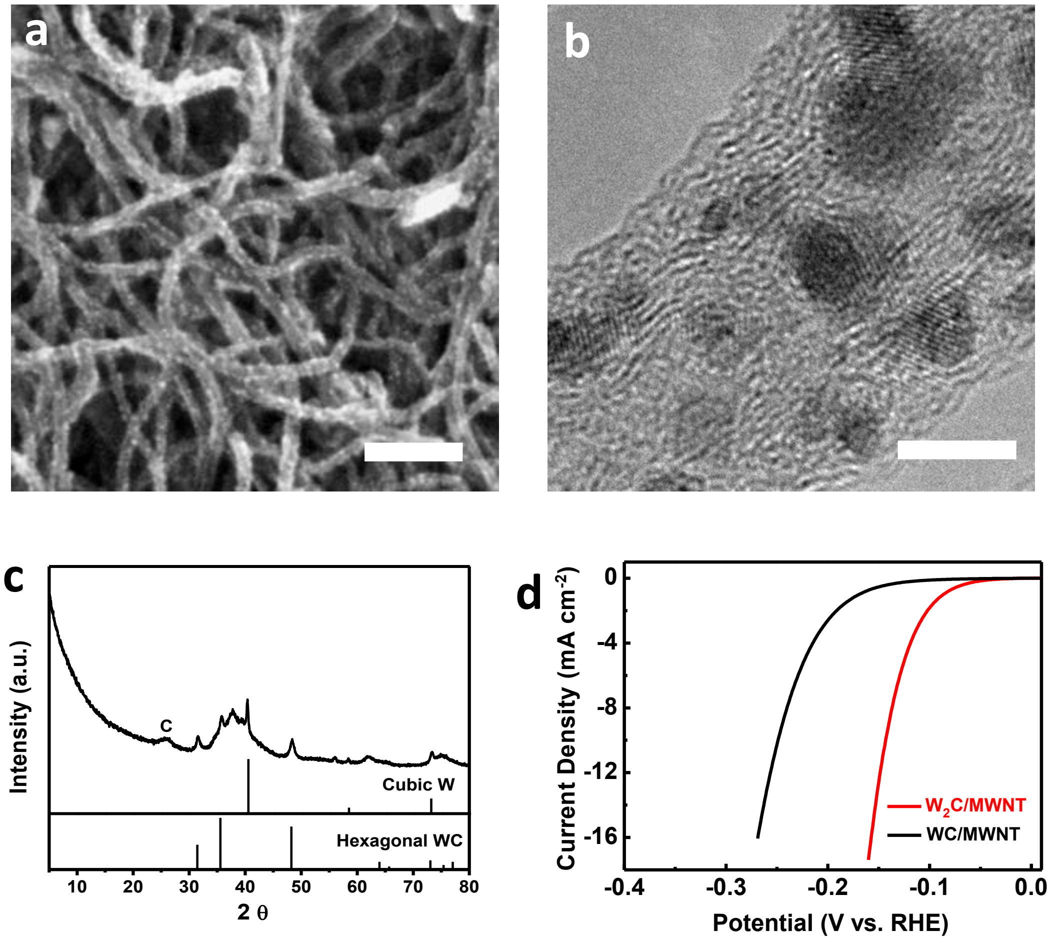

Supplementary Figure 4. WC/MWNT produced by the carburization reaction under CH_4 and its HER activity. (a) SEM image (scale bar, 100 nm), (b) TEM image (scale bar, 5 nm), (c) XRD pattern and (d) the polarization curves of WC/MWNT in comparison with W_2C/MWNT at a catalysts loading of 0.278 mg cm^{-2}.

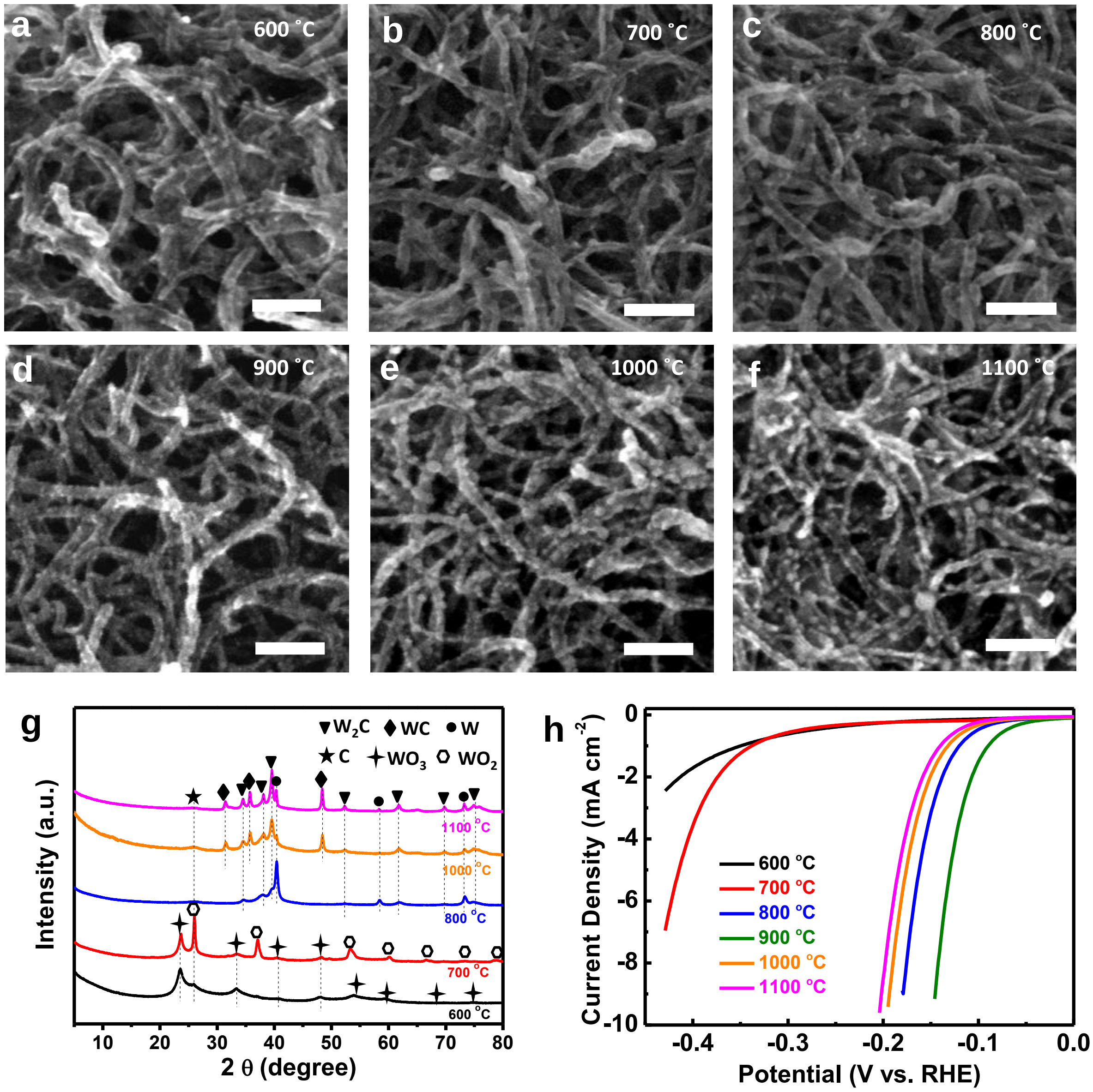

Supplementary Figure 5. The influence of different carburization temperatures on the microstructure, composition and HER activity of the final products. (a-f) SEM images (scale bar, 100 nm), (g) XRD patterns and (h) the polarization curves of different products at a catalyst loading of 0.278 mg cm^{-2}. It is obvious that 900°C yields the most active product.

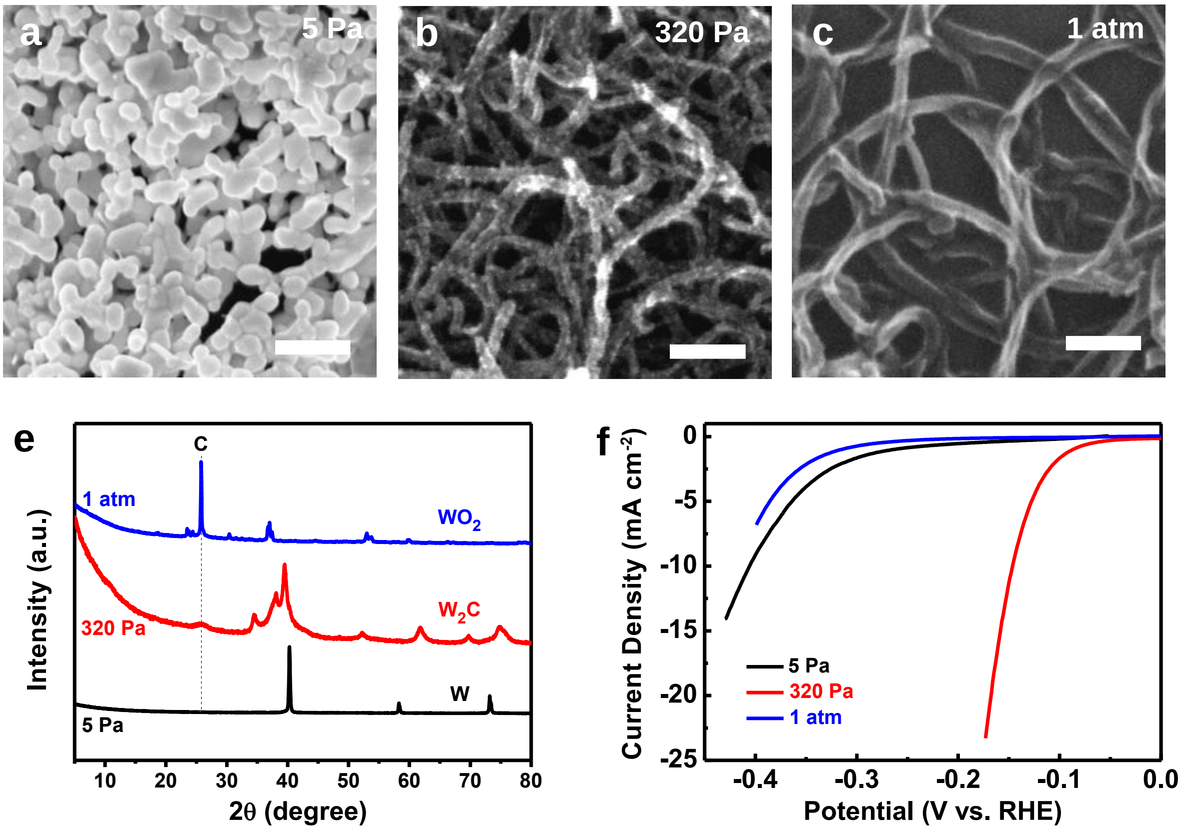

Supplementary Figure 6. The influence of different carburization pressures on the microstructure, composition and HER activity of the final products. (a-c) SEM images (scale bar, 100 nm), (e) XRD patterns and (f) the polarization curves of different products at a catalyst loading of 0.278 mg cm^{-2}. It is obvious that 320 Pa yields the most active product.

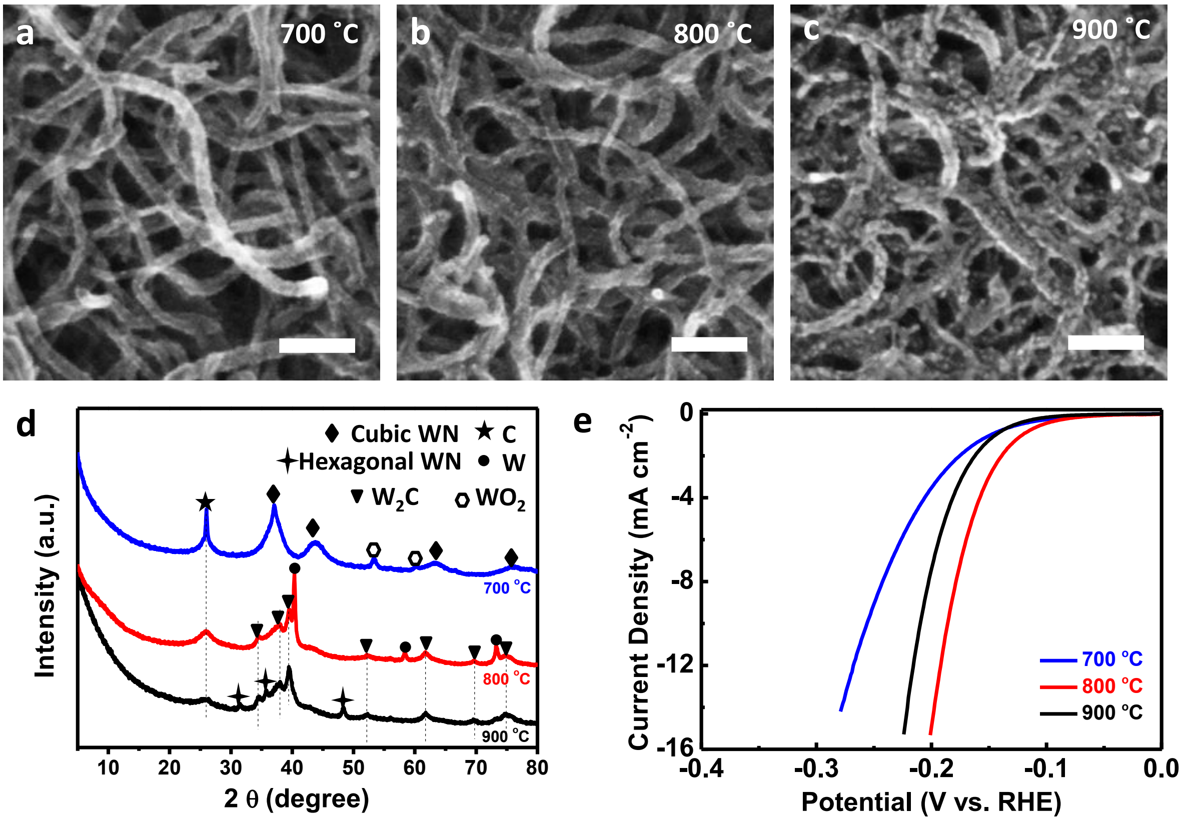

Supplementary Figure 7. Carburization products under NH_3 at different temperatures and their HER activities. (a-c) SEM images (scale bar, 100 nm), (d) XRD patterns and (e) the polarization curves of different products at a catalyst loading of 0.278 mg cm^{-2}.

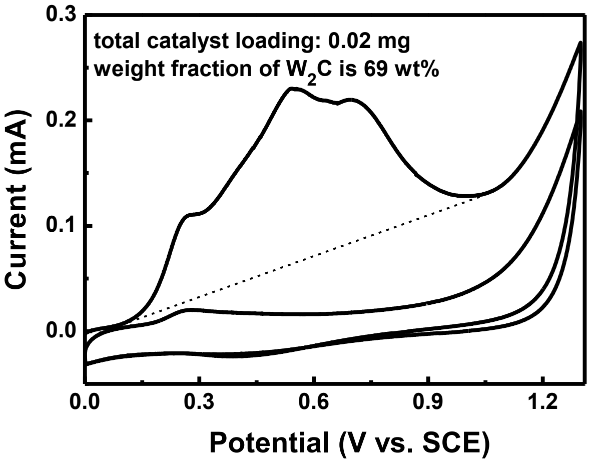

Supplementary Figure 8. CV curves of W_2C/MWNT in the anodic potential region exhibiting the irreversible oxidation to WO_3. The anodic waves were integrated to estimate the ECSA of W_2C. Integration of this oxidation envelope yields a total charge of 3.4×10^{-2} C, corresponding to 2.20×10^{-8} mol of W_2C on the electrode surface. At the meantime, we know the total catalyst loading on the working electrode is 0.02 mg, and ~69 wt% of it is W_2C. We therefore estimate that ~60% of W_2C in the composite is electrochemically accessible.

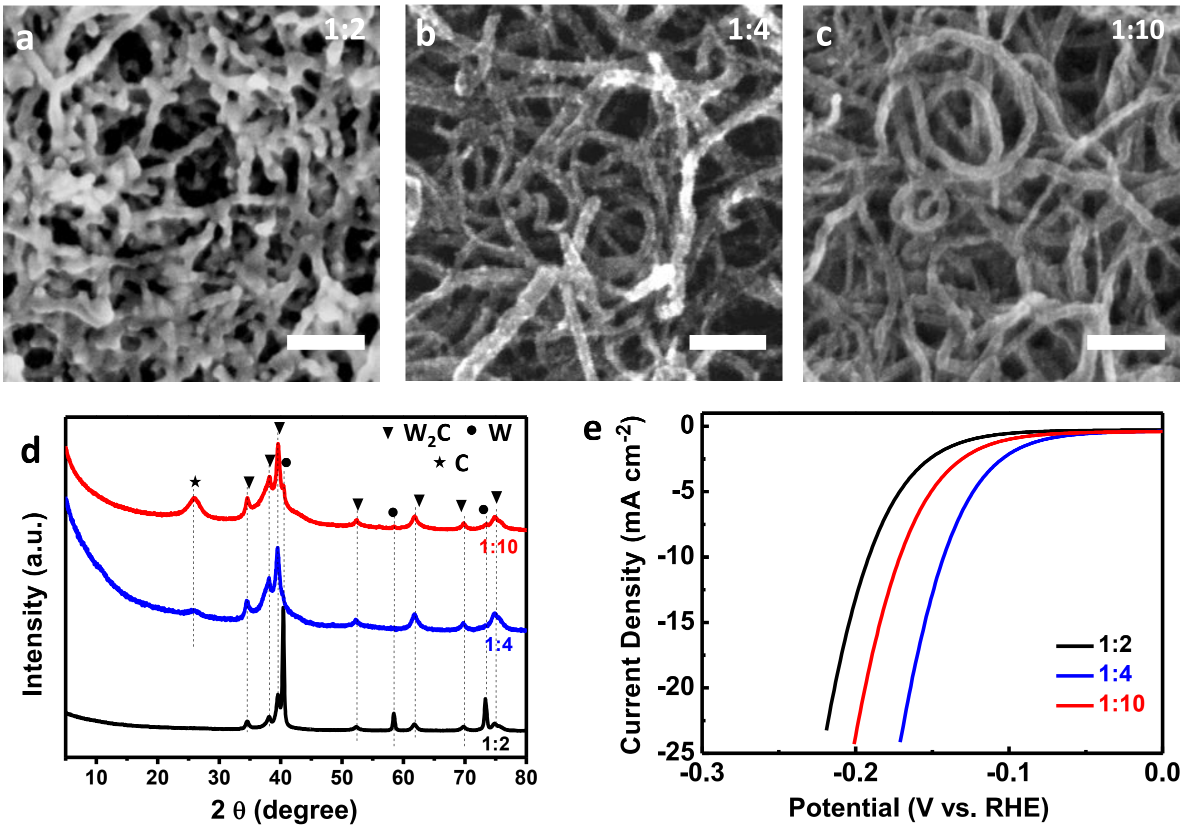

Supplementary Figure 9. The influence of different WCl_6 and MWNT (counted as C) starting molar ratios on the microstructure, composition and HER activity of the final products. (a-c) SEM images (scale bar, 100 nm), (d) XRD patterns and (e) the polarization curves of three different products at a catalyst loading of 0.278 mg cm^{-2}. It is obvious that WCl_6: MWNT = 1:4 yields the most active product.

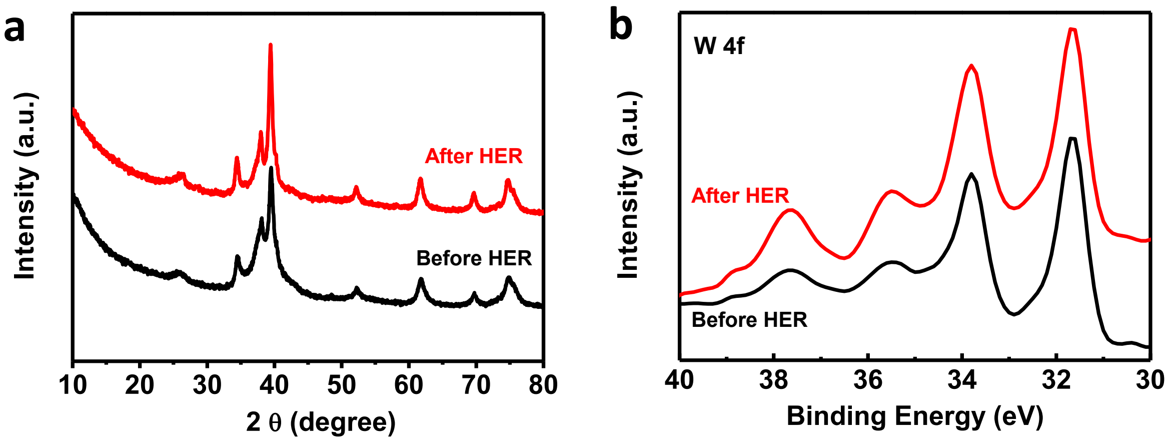

Supplementary Figure 10. (a) XRD patterns and (b) W 4f XPS spectra of W_2C/MWNT before and after the HER experiment. To prepare samples for the postmortem studies, catalyst powder was loaded onto carbon fiber paper electrode to achieve a high loading of 1 mg cm^{-2}, and subjected to continuous 1000 CV cycles. Then, catalyst powder was sonicated off the electrode and recovered for subsequent XRD and XPS studies.

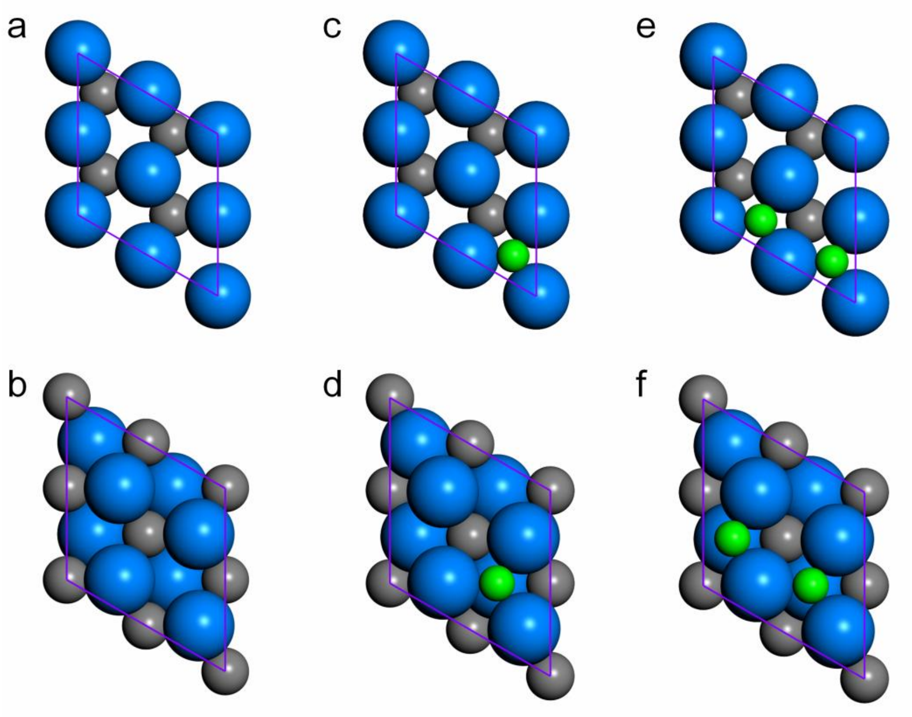

Supplementary Figure 11. Top views of geometric structure of (a) WC (0001), (b) W_2C (0001), (c) 0.25 ML_H on WC (0001), (d) 0.25 ML_H on W_2C (0001), (e) 0.5 ML_H on WC (0001) and (f) 0.5 ML_H on W_2C (0001)

Supplementary Table 1. A survey of W_2C synthetic methods reported in literature.

**Literature**	**Methods and Precursors**	**Compositions**	**Particle size**
J. Mater. Sci. **1993**, *28*, 5543-5547	CVD: SiH_4+ CH_4 + WF_6+ H_2, 1400 ˚C	SiC-W_2C composite	18~30 nm
J. Mater. Res. **1993**, *8*, 2702-2708	CVD: WCl_6+ C_2H_2+ H_2, 1000-1200 ˚C	α-WC + β-W_2C	0.1 ~0.3 µm
J. Phys. Chem. C **2013**, *117*, 3389−3395	HWCVD: W + TMDSCB, 2400 ˚C	W_2C	filaments
Mater. Transact. **2008**, *49*, 1256-1261	Reactive hot pressing: W + WC, 1600-1900 ˚C	W_2C	bulk
Mater. Transact. **2011**, *52*, 124-126	Reaction in molten salt: W + C, 1600-1900 ˚C	W_2C + W	0.3 ~0.5 µm
J. Am. Chem. Soc. **2012**, *134*, 3025−3033	Magnetron sputtering: W foil + WC, 825 ˚C	W_2C	thin film
J. Power Sources, **2012**, *202*, 11-17	Magnetron sputtering: W foil + WC, 825 ˚C	W_2C	thin film
Int. J. Hydrogen Energy **2008**, *33*, 6865-6872	AMT + resorcinol formaldehyde polymer, 900 ˚C	W_2C	2 ~ 4 µm microspheres
Angew. Chem. Int. Ed. **2005**, *44*, 6557-6560	AMT + resorcinol formaldehyde polymer, 900 ˚C	W_2C + WC + WC_{1-x}	2 ~ 4 µm microsphere
Int. J. Hydrogen Energy **2012**, *37*, 18591-18597	Plasma reaction: WO_3+ CH_4	WC (major) + W_2C (minor)	≤ 20 nm
Angew. Chem. Int. Ed. **2014**, *53*, 5131 –5136	silica-encapsulated WO_x NPs + CH_4/H_2, 775 ˚C	SiO_2/W_2C	nanoparticles

Supplementary Table 2. A survey of the HER performances of transition metal carbide based electrocatalysts from recent literatures.

Reference	Materials	Catalyst loading (mg cm^{-2})	Electrolyte	η @ *j* = 10 mA cm^{-2} (mV)	Tafel slope (mV dec^{-1})
This work	W_2C/MWNT	0.556	0.5 M H_2SO_4	123	45
**Angew. Chem. Int. Ed.* **2014**, *53*, 5131 –5136	α-WC/CB	0.724	0.5 M H_2SO_4	260	N/A
**ChemSusChem* **2013**, *6*, 168–181	WC NPs	1	0.5 M H_2SO_4	125	84
**Int. J. Hydrogen Energy* **2008**, *33*, 6865-6872	W_2C microspheres	N/A	1 M H_2SO_4	~170	118
**J. Am. Chem. Soc.* **2012**, *134*, 3025−3033	Thin film W_2C	N/A	0.5 M H_2SO_4	>300	69
	Thin film WC				91
**Angew. Chem. Int. Ed.* **2012**, *124*, 12875-12878	Mo_2C	1.4	1 M H_2SO_4	210	56
			1 M KOH	190	54
**Energy Environ. Sci.* **2014**, *7*, 387-392	Nanoporous Mo_2C NWs	0.21	0.5 M H_2SO_4	130	53
Energy Environ. Sci. **2013**, *6*, 943–951	Mo_2C/CNT	2	0.1 M $HClO_4$	152	55.2
**Chem. Commun.* **2014**, *50*, 13135–13137	Mo_2C/graphene	0.285	0.5 M H_2SO_4	~175	54
**ACS Nano* **2014**, *8*, 5164–5173	Mo_2C NPs on carbon nanotube-graphene	0.65	0.5 M H_2SO_4	~150	58
Energy Environ. Sci. **2014**, *7*, 387–392	np-Mo_2C NWs	0.21	0.5 M H_2SO_4	~135	~60
**Angew. Chem. Int. Ed.* **2015**, *54*, 1-6	Hierarchical β-Mo_2C	0.75	0.5 M H_2SO_4	172	62
			0.1 M KOH	112	55
Angew. Chem. Int. Ed. **2015**, *54*, 10752-10757	Mo_2C@NC	0.28	0.5 M H_2SO_4	124	60
**Angew. Chem. Int. Ed.* **2015**, *54*, 14723-14727	Mo_2C NPs@N-doped graphene	0.25	0.5 M H_2SO_4	78	41
**J. Mater. Chem. A* **2015**, *3*, 15505–15512	Mo_2C/CNT	8.2	0.1 M $HClO_4$	250	251
	Mo_2C/CXG	6.3		170	264
Chem. Sci. **2016**, *7*, 3399–3405	MoC-Mo_2C	0.14	0.5 M H_2SO_4	126	43
Chem. Commun. **2015**, *51*, 8323–8325	MoC/G	0.8	0.5 M H_2SO_4	221	88
	Mo_2C/G			150	57
**J. Am. Chem. Soc.* **2015**, *137*, 15753–15759	Mo_xC-Ni@NCV	1.1	0.5 M H_2SO_4	75	45

*Nature Commun. **2016**, *7*, 11204.	Mo_2C@NPC/NPRGO	0.14	0.5 M H_2SO_4	34	33.6

*Data reported in these works are questionable because they used Pt as the counter electrode, and their catalysts might be contaminated with Pt.

Supplementary Table 3. A survey of the PEC performances of Si nanowire based photocathodes from literatures.

**Reference**	**Photocathode**	**Electrolyte**	**Photocurrent density @ 0 V (mA cm^{-2})**	**Saturation photocurrent density (mA cm^{-2})**
This work	p-Si NWs/W_2C	0.5 M H_2SO_4	16	~30
J. Am. Chem. Soc. **2015**, *137*, 7035−7038	a-Si/Mo_2C	0.1 M H_2SO_4	11.2	~11.5
		1 M KOH	11.5	~12
Angew. Chem. Int. Ed. **2012**, *51*, 9128 –9131	MoS_x-Ti-n^+p-Si	1 M $HClO_4$	17	17
	Pt-Ti-n^+p-Si		15	15
ACS Nano **2014**, *8*, 8121-8129	$Ni_{12}P_5$/Si NWs	0.5 M H_2SO_4	21	22.5
Energy Environ. Sci. **2012**, *5*, 9653-9661	Ni–Mo/n^+p-Si MW	pH 4.5 KHP	9.1	9.1
Nature Mater. **2011**, *10*, 434-438	Mo_3S_4/Si pillar	1 M $HClO_4$	9.45	37
